# Supplementary figures and images for: An updated end-to-end ecosystem model of the Northern California Current reflecting ecosystem changes due to recent marine heatwaves
Source: PLoS One. 2024 Jan 19;19(1):e0280366. doi: 10.1371/journal.pone.0280366 (PMC10798527; doi:10.1371/journal.pone.0280366)

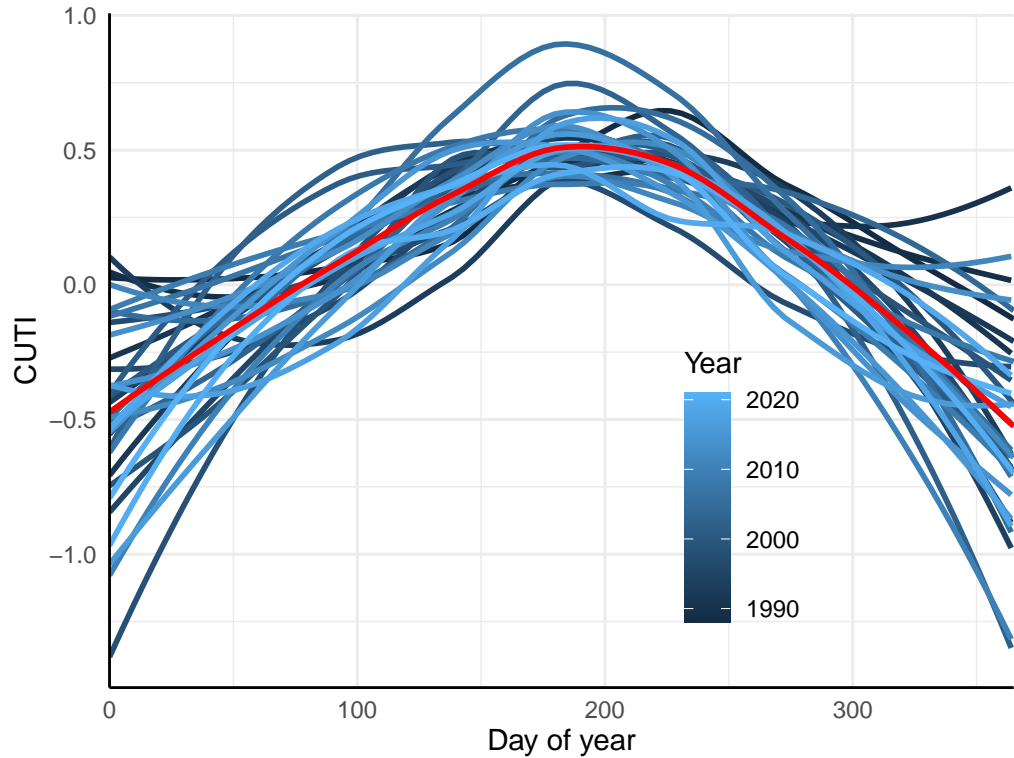

Supplement: S1 Fig — The Coastal Upwelling Transport Index (CUTI) is plotted on the y-axis against the day of year (x-axis). Each year (1988–2021) is plotted as an individual line in the blue gradient. The full timeseries (1988–2021) is used to drive the model for comparisons to the vertically generalized production model (VGPM) in Fig 10. The red line in the middle is the average CUTI time series (averaged by day of year across all years), which is used to drive the validation plot in Fig 9. (PDF) [file pone.0280366.s001.pdf]
