# Supplementary material for: An updated end-to-end ecosystem model of the Northern California Current reflecting ecosystem changes due to recent marine heatwaves
Source: PLoS One. 2024 Jan 19;19(1):e0280366. doi: 10.1371/journal.pone.0280366 (PMC10798527; doi:10.1371/journal.pone.0280366)
Supplement: S1 Appendix — (DOCX) [file pone.0280366.s008.docx]

# Appendix

## Biomass sources

### Coastal Pelagic Species

The SWFSC coastal pelagic species (CPS) acoustic-trawl survey is a north-to-south coastwide survey that pairs backscatter data from a multi-frequency echosounder and nighttime trawls to provide biomass estimates for Pacific sardine, Pacific herring, Pacific chub mackerel, Pacific jack mackerel, and northern anchovy [1, 2]. The CPS survey sampling domain fully covers the spatial domain of EcoTran (in both latitude and bathymetry). We used data from 2015-2019 to parameterize sardine, anchovy, herring, jack mackerel, and chub mackerel.

### Pacific Hake Ecosystem Acoustic Trawl Survey

The NWFSC conducts a joint US-Canada coastwide acoustic-trawl survey for Pacific hake (hereafter hake survey) during July and August in odd years (e.g., 2015, 2017). The hake survey sampling domain fully covers the spatial domain of EcoTran (in both latitude and bathymetry). The survey pairs fisheries acoustics data from multi-frequency echosounders with midwater trawls to estimate Pacific hake biomass for stock assessment [3, 4], and uses frequency differencing and an inversion model to estimate euphausiid biomass for ecosystem monitoring and research [5, 6]. We used data from 2015, 2017, and 2019 to parameterize krill and hake biomasses.

### Juvenile Salmon and Ocean Ecosystem Survey (JSOES)

The Juvenile Salmon and Ocean Ecosystem Survey is a long-term (1998-present) survey, which provided data on the pelagic community composition for salmonids, zooplankton, and seabirds from daytime surveys of the shelf off Oregon and Washington (between 44.25°N and 48.23°N) each year during May, June, and/or September [7, 8]. JSOES does not extend to the southern-most subregions of the EcoTran domain. Yet, it consistently samples within all three of our cross-shelf bathymetric zones. Juvenile and adult salmon and jellies were collected during daylight hours using a Nordic 264 pelagic rope trawl with a mouth opening of 30 m wide and 20 m height with a 0.8 cm cod-end liner. The net was typically towed for 30 minutes at the surface with a ship speed of ~6 km h^-1^ (3.2 knots).

Zooplankton were sampled via bongo tows and vertical nets [9, 10]. A 0.60 m diameter Bongo net (equipped with two black 335 µm mesh nets), is used to conduct double oblique plankton tows. The bongo net is fished such that a maximum depth of 20 to 30 m is reached. For the vertical net samples, a 0.5 m ring net (202 µm mesh) is used to collect a vertical plankton tow from 100 m, or 5 m from the bottom in shallower locations. For both zooplankton nets, flow meters are used to measure the volume of water filtered through the nets so that the prey densities can be calculated.

Seabird abundance and distributions were estimated from visual detections of birds within a bow-to-beam, 300 m wide arc (strip-transect method) [11, 12]. To avoid having trawl activity affect bird distributions, surveys began at dawn ~ 33 km offshore of the inshore-most sampling station, prior to any fishing operations. A trained observer used binoculars (8 x 42 mm) to detect, count, and identify birds within the survey strip. Each bird detection was recorded with a date, time, and position stamp into the custom “SeeBird” data acquisition software (NMFS Southwest Fisheries Science Center, La Jolla, CA).

### Newport Hydrographic Line

The Newport Hydrographic Line is a long-term, single cross-shelf transect survey off the central Oregon coast [13, 14], which occurs year-round at bi-weekly to monthly intervals. Observations from the survey allowed us to parameterize small invertebrate larvae and invertebrate (copepod and euphausiid) egg biomass. Mesozooplankton and egg densities are from two stations located on the inner shelf and outer shelf in 60 m and 300 m of water respectively. Smaller zooplankton were collected with a 0.5 m net fitted with 200 um mesh and hauled vertically from 100 m (or 5 m off the seafloor in depths shallower than 100 m). The Newport Hydrographic Line also provided data allowing us to partition the biomass of phytoplankton (from satellite imagery, described in main text) into large and small phytoplankton groups (see Table 2) and euphausiids (from the hake survey, described above) into the two dominant euphausiid species in the NCC, *Thysanoessa spinifera* and *Euphausia pacifica.* Biomass of the two krill species, *T. spinifera* and *E. pacifica*, were collected at night with a 0.6 m net towed obliquely over the upper 30 m of the water column at three stations located on the inner shelf and outer shelf in 60 m, 80 m, and 300 m of water.

### Pre-recruit survey

The Pre-recruit survey is a midwater trawl survey that occurs annually (2011, 2013-2019, 2022) around May-July along transects located along the Washington, Oregon, and Northern California coasts [15-17]. Stations are arrayed along cross-shelf transects set at every half degree of latitude both on and off the shelf. Stations are sampled cross-shelf past the EcoTran domain defined here, thus the Pre-recruit survey fully covers the spatial domain of EcoTran. A modified-Cobb midwater trawl with a 26 m headrope and a 9.5 mm cod-end liner is used to sample epipelagic micronekton at night with the headrope at around 30 m depth. This survey primarily samples micronektonic squid, shrimp, krill, larger gelatinous zooplankton, and mesopelagic and juvenile fish [18].

### West Coast Groundfish Bottom Trawl Survey

The NWFSC West Coast Groundfish Bottom Trawl Survey (hereafter groundfish survey) occurs annually from May-October, and uses a random stratified design to sample the entire west coast of the United States via a bottom trawl from 55-1280 m depths [19, 20]. Thus the groundfish survey fully covers the spatial domain of EcoTran. Data from 2003-2019 are available via the `nwfscSurvey` R package [21]. We used data from 2014-2019 to parameterize many groups of groundfish and crabs (see Table S1).

## Diet sources

### California Current Trophic Database

The California Current Trophic Database (https://oceanview.pfeg.noaa.gov/cctd/) was conceived and developed at the National Marine Fisheries Service (NMFS) Southwest Fisheries Science Center (SWFSC) in collaboration with trophic data contributors. The trophic database was compiled from twenty-four data sets, including stomach or scat contents, which were contributed from collaborators at the NOAA NMFS [SWFSC; NWFSC; and AFSC (Alaska Fisheries Science Center)], and academic institutions (University of California, Santa Cruz; Oregon State University; and Moss Landing Marine Laboratories). Once acquired, data were reviewed, standardized with communication and input from contributors, and synthesized. In total, diet composition data were obtained from 105,694 individual predators among 143 taxa collected throughout the California Current Large Marine Ecosystem from 1967–2019. These taxa consist of squids (n=5), elasmobranchs (n=13), bony fishes (n=118), and marine mammals (n=7).

The trophic database was created using the Structured Query Language (SQL) and consists of eight data tables. Hierarchical (parent-child) tables include Data Sources, Collection Information, Predator Information, Prey Composition, and Prey Size. The trophic database contains two taxonomic tables, one for predators (n=143) and one for prey (n=1659), that are linked to the Predator Information and Prey Composition tables. The World Register of Marine Species (WoRMS; https://www.marinespecies.org/) was primarily used for taxonomic reference, and scientific names from each data set were updated accordingly. Additional taxonomic resources (e.g., recent primary literature, California Academy of Fishes Fish Catalog) were incorporated when WoRMS designations seemed dated or unsubstantiated. A single flat file that was created from the five hierarchical SQL tables is provided to the public via SWFSC’s ERDDAP site, along with Glossary, Predator Taxonomy, and Prey Taxonomy tables. Predator diets were processed as described in the main text.

### Juvenile salmon diets

Juvenile salmon stomach content data were collected from the JSOES survey (see survey description above). Prey items were removed from stomachs and identified to the lowest possible taxonomic level, counted, and weighed to the nearest 1 mg. Salmon with empty stomachs were removed from the analysis since these data are uninformative for parameterizing diet contributions. Similarly, since we broke out juvenile Chinook salmon into multiple functional groups (Table 2; see “Salmonids” subheading in main text) based on genetic stock information, those observations without associated genetic stock information were removed as they are also unable to inform the diets of our various salmon functional groups. All unidentified prey material and non-living material (i.e., plastic) were also removed from the diets. Then we calculated the relative contribution of each species or taxonomic group as prey (allocated to and aggregated by our functional groups in Table 2) to each of the juvenile salmonid functional groups by summing the weights of prey groups and dividing by the total weight of prey consumed for that juvenile salmonid group (see supplementary code and diet matrix for more information).

**References**

1. Zwolinski JP, Demer DA, Cutter Jr GR, Stierhoff K, Macewicz BJ. Building on fisheries acoustics for marine ecosystem surveys. Oceanography. 2014;27: 68–79.

2. Stierhoff KL, Zwolinski JP, Demer DA. Distribution, Biomass, and Demography of Coastal Pelagic Fishes in the California Current Ecosystem During Summer 2019 Based on Acoustic-Trawl Sampling. US Department of Commerce, NOAA Technical Memorandum NMFS-SWFSC-626. 2020. doi:10.25923/nghv-7c40

3. Fleischer G, Cooke KD, Ressler PH, Thomas RE, De Blois SK, Hufnagle LC. The 2005 integrated acoustic and trawl survey of Pacific hake, Merluccius productus, in US and Canadian waters off the Pacific coast. 2008.

4. de Blois S. The 2019 Joint US–Canada Integrated Ecosystem and Pacific Hake Acoustic-Trawl Survey: Cruise Report SH-19-06. 2020.

5. Chu D, Lawson GL, Wiebe PH. Estimation of biological parameters of marine organisms using linear and nonlinear acoustic scattering model-based inversion methods. The Journal of the Acoustical Society of America. 2016;139: 2885–2895.

6. Phillips EM, Chu D, Gauthier S, Parker-Stetter SL, Shelton AO, Thomas RE. Spatiotemporal variability of euphausiids in the California Current Ecosystem: insights from a recently developed time series. ICES Journal of Marine Science. 2022;79: 1312–1326.

7. Brodeur RD, Fisher JP, Emmett RL, Morgan CA, Casillas E. Species composition and community structure of pelagic nekton off Oregon and Washington under variable oceanographic conditions. Marine Ecology Progress Series. 2005;298: 41–57.

8. Peterson WT, Morgan CA, Fisher JP, Casillas E. Ocean distribution and habitat associations of yearling coho (Oncorhynchus kisutch) and Chinook (O. tshawytscha) salmon in the northern California Current. Fisheries Oceanography. 2010;19: 508–525.

9. Schabetsberger R, Morgan CA, Brodeur RD, Potts CL, Peterson WT, Emmett RL. Prey selectivity and diel feeding chronology of juvenile chinook (Oncorhynchus tshawytscha) and coho (O. kisutch) salmon in the Columbia River plume. Fisheries Oceanography. 2003;12: 523–540.

10. Morgan CA, De Robertis A, Zabel RW. Columbia River plume fronts. I. Hydrography, zooplankton distribution, and community composition. Marine Ecology Progress Series. 2005;299: 19–31.

11. Heinemann D. A range finder for pelagic bird censusing. Journal of Wildlife Management. 1981;45: 489–493.

12. Tasker ML, Jones PH, Dixon TIM, Blake BF. Counting seabirds at sea from ships: a review of methods employed and a suggestion for a standardized approach. The Auk. 1984;101: 567–577.

13. Peterson WT, Miller CB. Year-to-year variations in the planktonology of the Oregon upwelling zone. Fishery Bulletin. 1975;73: 642.

14. Peterson WT, Keister JE, Feinberg LR. The effects of the 1997–99 El Niño/La Niña events on hydrography and zooplankton off the central Oregon coast. Progress in oceanography. 2002;54: 381–398.

15. Miller RR, Santora JA, Auth TD, Sakuma KM, Wells BK, Field JC, et al. Distribution of pelagic thaliaceans, *Thetys vagina* and *Pyrosoma atlanticum*, during a period of mass occurrence within the California Current. CalCOFI Rep. 2019;60: 94–108.

16. Brodeur RD, Auth TD, Phillips AJ. Major shifts in pelagic micronekton and macrozooplankton community structure in an upwelling ecosystem related to an unprecedented marine heatwave. Frontiers in Marine Science. 2019;6: 212.

17. Northwest Fisheries Science Center. Juvenile Fish Data - Coastwide Cooperative Pre-recruit Survey. 2021. Available: https://www.fisheries.noaa.gov/inport/item/20562

18. Friedman WR, Santora JA, Schroeder ID, Huff DD, Brodeur RD, Field JC, et al. Environmental and geographic relationships among salmon forage assemblages along the continental shelf of the California Current. Marine Ecology Progress Series. 2018;596: 181–198.

19. Keller AA. The 2005 US West Coast bottom trawl survey of groundfish resources off Washington, Oregon, and California: Estimates of distribution, abundance, and length composition. NOAA Technical Memorandum. 2008.

20. Keller AA, Wallace JR, Methot RD. The Northwest Fisheries Science Center’s West Coast Groundfish Bottom Trawl Survey: history, design, and description. NOAA Technical Memorandum NMFS-NWFSC-136. 2017.

21. Wetzel CR, Johnson KF, Hicks AC. nwfscSurvey: Northwest Fisheries Science Center Survey. R package version 20. 2021. Available: https://rdrr.io/github/nwfsc-assess/nwfscSurvey/
