# Supplementary material for: An updated end-to-end ecosystem model of the Northern California Current reflecting ecosystem changes due to recent marine heatwaves
Source: PLoS One. 2024 Jan 19;19(1):e0280366. doi: 10.1371/journal.pone.0280366 (PMC10798527; doi:10.1371/journal.pone.0280366)
Supplement: Attachment — Submitted filename: Gomes_et_al.pdf [file pone.0280366.s009.pdf]

An updated end-to-end ecosystem model of the Northern California Current reflecting ecosystem changes due to recent marine heat waves

For consideration in PLOS One

Authors: Gomes et al.

Manuscript ID: PONE-D-22-35465

This manuscript presents what is essentially an updated version of an existing end-to-end model (Ruzicka et al.) of the Northern California Current ecosystem, with several technical modifications/improvements. Apparently, the impetus for the update (at this time) is the need to account for anomalous oceanographic conditions that occurred throughout the past decade, to facilitate better understanding of how those conditions may impact the ecosystem, including several vulnerable/endangered species (e.g., Pacific salmon).

It seems the intent of this manuscript was not to report the responses of the ecosystem and these vulnerable species to the changing ocean conditions, but instead to validate the ecosystem model by presenting several general, pseudo-quantitative metrics of model performance (relative to expectations), in addition to diagnostic criterion associated with large end-to-end models, as well as the data inputs. From this standpoint, the manuscript has succeeded and I have not found any deficiencies or methods that appear to be incorrect. Additionally, the documentation of the methods and input data was very clear, and everything was easily accessible, per the references in the text.

I will say that the predicted increase in southern resident killer whale abundance was a bit counterintuitive, given that it was one of only two groups to show a significant increase, and that the population has been is such a precarious state the past two decades. I suppose this could be the topic for further research using this updated model? All told, I only have several very minor corrections for the authors to address, which are listed below.

Major comments/issues

(None)

Minor comments/issues

Line 479: References here are 64 (which is bird-related) and 65; shouldn't this be 65 and 66 instead?

Line 565: Again, references here appear to be off, by one. I'm assuming you're referring to 69 and 70 here, not 68 and 69?

Lines 641-646: This sentence reads awkwardly in its current form. I recommend breaking it up, or using alternate punctuation.

Fig. 9: The red against black color theme of this plot may be difficult for colorblind readers to interpret—I suggest replacing red with purple, green, or yellow to go against the black.
