# Supplementary material for: An updated end-to-end ecosystem model of the Northern California Current reflecting ecosystem changes due to recent marine heatwaves
Source: PLoS One. 2024 Jan 19;19(1):e0280366. doi: 10.1371/journal.pone.0280366 (PMC10798527; doi:10.1371/journal.pone.0280366)
Supplement: Attachment — Submitted filename: Response to reviewers.docx [file pone.0280366.s010.docx]

To whom it may concern,

We appreciate that both reviewers have done a great job assessing the details of the 80-page manuscript and the 1,500 files of the data/code repository that was included. We believe that both reviewers see the value in this submission and offer suggestions for improvement, and that these concerns have been fully addressed here and have certainly improved the clarity of our manuscript and data/code repository.

Thank you for your time and consideration and we look forward to your reply.

On behalf of all co-authors,

Dylan Gomes

Reviewers' comments:

Reviewer's Responses to Questions

**Comments to the Author**

1. Is the manuscript technically sound, and do the data support the conclusions?

The manuscript must describe a technically sound piece of scientific research with data that supports the conclusions. Experiments must have been conducted rigorously, with appropriate controls, replication, and sample sizes. The conclusions must be drawn appropriately based on the data presented.

Reviewer #1: Yes

Reviewer #2: Yes

2. Has the statistical analysis been performed appropriately and rigorously?

Reviewer #1: N/A

Reviewer #2: Yes

3. Have the authors made all data underlying the findings in their manuscript fully available?

The [PLOS Data policy](http://www.plosone.org/static/policies.action#sharing) requires authors to make all data underlying the findings described in their manuscript fully available without restriction, with rare exception (please refer to the Data Availability Statement in the manuscript PDF file). The data should be provided as part of the manuscript or its supporting information, or deposited to a public repository. For example, in addition to summary statistics, the data points behind means, medians and variance measures should be available. If there are restrictions on publicly sharing data—e.g. participant privacy or use of data from a third party—those must be specified.

Reviewer #1: Yes

Reviewer #2: Yes

4. Is the manuscript presented in an intelligible fashion and written in standard English?

PLOS ONE does not copyedit accepted manuscripts, so the language in submitted articles must be clear, correct, and unambiguous. Any typographical or grammatical errors should be corrected at revision, so please note any specific errors here.

Reviewer #1: Yes

Reviewer #2: Yes

5. Review Comments to the Author

Please use the space provided to explain your answers to the questions above. You may also include additional comments for the author, including concerns about dual publication, research ethics, or publication ethics. (Please upload your review as an attachment if it exceeds 20,000 characters)

Reviewer #1: An updated end-to-end ecosystem model of the Northern California Current reflecting ecosystem changes due to recent marine heat waves
For consideration in PLOS One
Authors: Gomes et al.
Manuscript ID: PONE-D-22-35465

This manuscript presents what is essentially an updated version of an existing end-to-end model (Ruzicka et al.) of the Northern California Current ecosystem, with several technical modifications/improvements. Apparently, the impetus for the update (at this time) is the need to account for anomalous oceanographic conditions that occurred throughout the past decade, to facilitate better understanding of how those conditions may impact the ecosystem, including several vulnerable/endangered species (e.g., Pacific salmon).

It seems the intent of this manuscript was not to report the responses of the ecosystem and these vulnerable species to the changing ocean conditions, but instead to validate the ecosystem model by presenting several general, pseudo-quantitative metrics of model performance (relative to expectations), in addition to diagnostic criterion associated with large end-to-end models, as well as the data inputs. From this standpoint, the manuscript has succeeded and I have not found any deficiencies or methods that appear to be incorrect. Additionally, the documentation of the methods and input data was very clear, and everything was easily accessible, per the references in the text.

Thank you for your comments and positive feedback.

I will say that the predicted increase in southern resident killer whale abundance was a bit counterintuitive, given that it was one of only two groups to show a significant increase, and that the population has been is such a precarious state the past two decades. I suppose this could be the topic for further research using this updated model? All told, I only have several very minor corrections for the authors to address, which are listed below.

Thank you for this comment. We do not actually predict an increase in SRKW over time, instead we are just looking at stability in the production of those groups over time. The fact that most groups asymptote when run with this constant average upwelling timeseries only suggests that the model is stable, not that we would predict all of these groups to remain the same over a real timeseries with strong inter-annual variation. Marine mammals are so long-lived that this asymptote can take a very long time to reach, which is what is occurring for SRKWs in this figure. This is perhaps confusing and is clarified in the manuscript on lines 664-678 and the Fig 9 caption. Additionally, we have extended the simulation to 150 years (instead of 100), to show that all groups reach stability, which should aid in preventing other readers from being confused by the highlighted increase in SRKWs.

Major comments/issues
(None)

Minor comments/issues
Line 479: References here are 64 (which is bird-related) and 65; shouldn’t this be 65 and 66 instead?
Line 565: Again, references here appear to be off, by one. I’m assuming you’re referring to 69 and 70 here, not 68 and 69?

Thank you for catching these mistakes. Our citation manager for some reason had not updated the numbers in the references prior to submission, and made some the references off by a value of 1. We have now fixed this and appreciate your attention to detail.

Lines 641-646: This sentence reads awkwardly in its current form. I recommend breaking it up, or using alternate punctuation.

Thank you for this point. We agree this sentence was clunky and confusing, and have clarified this in the revised manuscript, now on lines 657-662.

Fig. 9: The red against black color theme of this plot may be difficult for colorblind readers to interpret—I suggest replacing red with purple, green, or yellow to go against the black.

Thank you for this point. I was only aware of the red-green distinction being difficult for colorblind readers. We have now changed this in our updated manuscript.

Reviewer #2: A Review of “An updated end-to-end ecosystem model of the Northern California Current reflecting ecosystem changes due to recent marine heat waves”

Here the authors present an updated and expanded ecosystem model that focusses on food web dynamics of the Northern California Current. The model is updated from a previously published version to include greater spatial domain, additional functional groups and greater resolution of some species groups (with a focus on endangered Chinook salmon), and to represent a more current time period (2014-2019) that was characterized by marine heatwave conditions. The model is driven by upwelling time series that drives nutrient input and thus primary production in the model. The model is presented as a tool for future analyses of food web dynamics, and specifically Chinook salmon trophic dynamics, under climate change scenarios.

This manuscripts describes the development of a tool (a model) and fits under the PLOS ONE submission category of “new methods, software, databases, or tools“. The Journal guidelines refer to acceptance criteria for this type of submission as (1) Utility, (2) Validation, and (3) Availability. I reviewed the manuscript accordingly.

I have reservations about recommending this manuscript for publication. I believe the model is sound and will support ecosystem analyses that will be submitted for future publication. However, the manuscript does not adequately fulfill the three categories to be published independently as a new tool. I would welcome its publication accompanied by analyses using the model (with most model details in the appendix), or conversely, additional development (and resubmission) with more of a focus of making the model a usable and available tool for other scientists and managers.

Thank you for your positive feedback. We have prepared a resubmission that focuses more on making the model a “usable and available tool”, by adding additional details and clarification to the data/code repository (your second suggested option). However, we also believe that criteria within your first suggested option have already been met; the manuscript already does include quite some analyses using the model (Figs 4-10 are model outputs, and additional new Figs 11-13 are as well), and we want to be careful not to make the 80-page manuscript even more cumbersome than it already is by including too many additional analyses.

Utility
1. Use as a tool:
a. This model will be useful in examine trophic dynamics in the NCC, with a focus on Chinook salmon. The relatively large number of Chinook functional groups makes this model well-suited to salmon related ecosystem analyses.

Thank you for your positive feedback.

b. The model was updated to represent the 2014-2021 marine heatwave period and potential changes in ecosystem state. I am not sure how the model will be used to show these potential changes, as there is no equivalent model in a previous time period for comparison. The previously published model would have a reduced footprint and functional groups, and no separation of Chinook salmon, potentially challenging the interpretation of differences in model outputs. Please note my point #2 under the Validation section for further discussion. I acknowledge the difficulty in assessing the ‘utility’ of this model without a detailed discussion of how the authors plan to use it.

Thank you for your comments. We do not need a second “equivalent” model to assess changes in the ecosystem state. Previous work has been published on the ecosystem that we can directly compare to this ecosystem model and its outputs (e.g., many of these values are expressed per unit area, or grouped by functional group aggregates that were combined for analysis). Additionally, most of the functional groups are identical across models. As far as functional group changes go, only minor modifications have been made to improve species resolution around Chinook salmon and marine mammals to make modelling scenarios more useful for endangered and commercially fished Chinook salmon. This doesn’t mean that the two models cannot be directly compared for all other 90+ functional groups (as well as an aggregated Chinook or killer whale group). We believe we have suggested possible uses of the model on lines 828-834 (now lines 866-872): “The EcoTran ecosystem modeling framework can be used to estimate the pressure that various consumer groups exert on lower trophic levels and the rest of the ecosystem, identify important food web nodes and how energy is transferred between them, compare ecosystem states during periods of low and high predator or competitor biomass, and to conduct simulation analyses to estimate the impact of events such as northward expansions of fish, jellyfish blooms, fishing pressure, climate change, and other events upon the ecosystem [32,32–34,77,115].”, but agree that to be more specific than this would be to clarify exactly how we plan to use it in the near future, which would have little utility in this manuscript.

c. In considering the ‘Utility’ of the model beyond the immediate author group, I believe it will be challenging. In my opinion, it is not well enough presented and supported to have utility beyond people with intimate knowledge of the model. While I applaud the large effort to document and make publicly available the data files and code to produce this model (Zenodo repository), I do not believe it is easily and readily available ‘Tool’ for use by other scientists and managers, limiting its utility.

Thank you for your comments and positive feedback about the large effort to document and make available the files and code. We agree that as the repository was, it was quite confusing as to how to use the model. We have now separated the repository into “reproducibility”, which describes how we built the model (not necessary for future uses of the model), and “ECOTRAN_Code”, which describes the code and final model file (which completely allows the user to build their own model scenarios and simulations relatively easily). We have now also included a 22-page README about the model code that we accidentally omitted originally. Future users will only have to edit the first few lines of one MATLAB script in order to use the model for an infinite number of model scenarios and simulations – this has now been clarified in the data and code repository.

I will note this level of publicly available data files exceeds the documentation and availability of most models used to support published analyses of trophic dynamics. An advanced level of model utility and availability is not a requirement in those scenarios. But if the model is being published independently as a new ‘Tool’ then I believe more could be done to allow for its use by a broader audience.

Thank you for your comments. This submission isn’t solely a new ‘Tool’, but also includes many model outputs, many of which are already usable to others working in the ecosystem (Figs 4-10, and recently added Figs 11-13, all Tables, supplementary data, etc.). We have now further clarified the use of the model and distinguished the many files (data & code) that belong to the reproducibility part of the submission (which are not required to use the tool). See comments above about improvements to the documentation, including the 22-page readme for the model code. While we have made clarifying changes to the new version of the repository, we also recognize that more can always be done to improve ease of use. The manuscript itself is already 80 pages and the data/code repository 1500 files. We have added additional clarifying details now to the supplemental data/code repository, which we agree will make it more useful.

Some examples to support this comment:
i. The “README.txt” file states how there is no workflow to follow from start to finish, some data are not provided due to data ownership criteria, and some files may be missing.

This isn’t exactly true. It states “there isn't a **completely linear** workflow to get from start to finish”. This is an important distinction, as the workflow is there, but it is somewhat iterative. Importantly, this is the workflow for the reproducibility part of the data/code submission, not the use of the model, which has now been more thoroughly detailed in a revised data/code submission. We have also more properly highlighted the two distinct parts (reproducibility and model use) of the data/code repository, which should lead to less confusion. Some raw data that were used to build the model are only available here as a header with a few rows. This is due to data contributors being unwilling to openly share data. This, however, does not change the usability or availability of the model, as these data are only necessary to reproduce the model from “scratch”. Thus, the files that call these header data files are included in the spirit of transparency and open science, which does not detract from anyone’s ability to use the tool.

ii. The main model file (“NCC2_08032022.csv”) is available and could be run but if other users wanted to modify the model for different applications, it would be challenging to work through the underlying files and code.

We agree that this would be challenging as it took us nearly 2 years to update this model for different applications than were previously available. This tool is built to be specifically for the Northern California Current. Any adaptation to other ecosystems will require substantial work, which is not limited by the data and code we’ve provided, but rather future user’s own time. Many of the underlying files and code are simply for the sake of reproducibility, and are not needed to use the model. This has now been clarified in the repository (see responses above). As it stands, the main model file you reference, and the code to run scenarios are immediately available and relatively easy to use, as the user only needs to open one MATLAB file and edit the first few lines of annotated code to run simulations – this has also been clarified in the newest version of the data and code repository. We have improved the language around this within our initial readme file to make this more clear, and appreciate you pointing this out to us.

iii. The calculations span more than one programming software

While this isn’t exactly merit for disqualification, again, this isn’t entirely true for the “tool” part of the model. The model was built using R and MATLAB code. So indeed, to **reproduce** our calculations, you would have to use both languages. However, the model is run purely on MATLAB code, so users can indeed use this tool with only one programming software.

iv. The “FlowChart.pdf” on Zenodo describes the workflow at a high level but not enough to easily recreate/modify the model.

We agree that the FlowChart.pdf doesn’t describe the workflow enough to recreate or modify the model, but this wasn’t the point of this document. The document was intended to do exactly as you say, show a “high level” description of what is going on – i.e., only to orient the user. The highly annotated code itself and individual readme files within each subdirectory are what provides the detail to recreate or modify the model. Additionally, as stated above, we left out an important 22-page document that provides the thorough details on the model code.

2. Diet updates:
a. I suggest the authors are more specific in how the model was updated and can be interpreted. For example in Line 161: “We focused on data collected primarily during and after recent marine heatwaves (2014 – 2021) to more accurately reflect the current conditions within the NCC, as there is evidence that the ecosystem has entered a novel state [15,16]. We updated the representation of food web interactions with published and unpublished datasets and reports to reflect potential reshuffling of trophic links.” The model more accurately reflects the biomass and catch of the MHW period, but less so of “reshuffling of trophic links” since the diets do not specifically reflect that time period (more on this point below). It would be interesting to include diet data from this time period if available (which is challenging to have available data), especially for Chinook salmon since they are the focus of the model.

We agree that we demonstrate the reshuffling of trophic links “less so” than biomass and catch, although we still do demonstrate the reshuffling of some trophic links, as is, because we have incorporated many newer diet studies, including from a long time series of juvenile Chinook salmon, which does change the strength and ties of the trophic connections (even with some of the original connections still included). We have been more careful in the wording, per your comments above and below. For example, we have specified that we only had updated diet information for 26 functional groups, 19 of which were during MHW years, “We updated the representation of food web interactions for 26 functional groups, including diet data from the MHW period for 19 groups, with published and unpublished datasets and reports to reflect potential reshuffling of some trophic links.” (Lines 166-169), and have added additional caveats and explanation in the Methods:

“Thus, it is important to note that the diet matrix does not fully represent changes since the onset of marine heatwaves (MHW). We were able to update the diets of 26 functional groups, 19 of which included samples from the MHW years (2014 and later; see Table S1). The addition of diets collected during the MHW period will reflect some shifts in diet trophic dynamics, even if averaged with older data. However, it will be more conservative in how far the diets have shifted since we are averaging them with their original diets. We think this conservative approach is beneficial because diet studies are stochastic (depending completely on when and where individuals are sampled) and have high degrees of uncertainty. In an ideal world, this updated model would include solely diet data from 2014 and onwards, yet decisions were ultimately driven by the fact that so few diet studies have been made available since the onset of marine heatwaves.” (lines 567-577).

We agree that these details are important to include.

b. While diet data were updated, it is difficult to determine which groups were updated and what time period they represent from Table S1. Following the links to the online diet database (<https://oceanview.pfeg.noaa.gov/cctd/> ; source of diet data for some groundfish), it lists the most current data as 2016 for Pacific herring and sardine, and 2011 for sablefish, as examples. It could be stated more clearly what time frame the diet data represent (newest surveys plus what older data were averaged together) to determine, for example, if the marine heatwave period is represented in the average or not.

Thanks for this comment. We agree that this would be a useful contribution, and have now included this as an additional column in the supplementary Table S1 in the latest revision.

c. Diet averaging methods: The authors describe their method of updating diet data as an average across all available diet data (including newer data and the original model data) to broaden the sources of information and reflect the diverse prey field (Line 549). I don’t disagree with this method but would suggest the authors highlight that ‘updating’ the diets does not reflect the MHW period specifically and would not reflect potential shifts in diet trophic dynamics.

Thank you for this point. We agree that we need to be more careful in how we describe this, and have edited the manuscript to make this more clear. See response above to point 2a for more details. In an ideal world with an unlimited supply of sampling effort, we would indeed only use MHW period diet data to parameterize the MHW period, but do not believe that to be feasible given the sparseness of diet studies.

Validation
1. The authors followed published and well-regarded prebalance validation methods described in Link (2010). These are legitimate steps in validating a model’s stability and basic functioning but do not speak to how well the model captures the dynamics of the specific system (Northern California Current) and trophic dynamics it is intended to represent. It is helpful to see the comparison of primary production estimates (Fig 10) as one step of this validation but the rest of the model’s trophic dynamics/biomass trajectories are not validated in that way. Comparisons to biomass time series is a common practice for models starting at an earlier time period, but given the more recent time period the model, I can see how that would be challenging. Heymans et al. (2016) describe “Best Practices for validation of Ecosim models”, which could also be useful.

Thank you for your comments and for the additional reference, which is now included in the updated manuscript. We agree that the prebalance validation methods are legitimate but don’t demonstrate how well the model captures the dynamics of the ecosystem. As no earlier papers using Ecotran do any timeseries validation (see Ruzicka et al. 2012, 2016a, 2016b, 2018; Chiaverano et al. 2018, referenced in the initial submission), we intended to go beyond the basic PREBAL steps by including the primary production timeseries, which, in turn, drives the rest of the consumption in the system. We have improved the manuscript by including comparisons (model output to biomass time series) of 7 other available taxa (new Figures 11 and 12), and we agree that this has greatly improved the manuscript.

2. I question why the expanded model was not initiated in the time period of the original published model (Ruzinka et al. 2010) so that it could be (1) compared to the original model with reduced functional groups and spatial footprint to determine how the expansion of the model changed the trophic dynamics and interpretation, (2) allow for a comparison of the MHW ecosystem structure with that portrayed in the earlier model (a stated purpose of this update) and (3) run forward in time and fit to time series of biomass and catch. In this way, it could be determined if the MHW conditions were captured by the model, it could be validated using the historic trends in biomass, and then run into the future based on that understanding.

Thank you for your comments. If we understand your question, you question why we didn’t build an additional model that expanded on the functional groups and spatial footprint of the original model (without updating the biomass and diets). Put quite simply, that would be months of additional work that wouldn’t give us a model that is updated for current conditions. While this comparison may be a useful modeling exercise, it doesn’t get us closer to having a management-ready model for our ecosystem as it currently is.

To point #1, expanding a few functional groups and increasing the spatial resolution shouldn’t be dramatically changing the trophic dynamics and interpretation of the model. Model currencies are expressed per unit area, so adding additional resolution doesn’t change the overall average trends and patterns, instead we are able to learn more about specific groups and specific locales.

To point #2, your suggested changes are not all necessary to make the comparison that you are suggesting. The energy budget and network properties that emerge are directly comparable across models without having to change the spatial extent of the models, which don’t differ dramatically. To compare across functional groups, collapsing to common denominators (aggregated juvenile Chinook groups, for example), are easy ways to make these comparisons without the additional steps of re-parameterizing and re-balancing an additional food web model. In fact, this is what we are currently doing for a direct comparison between models. However, this comparison is an additional effort that will be a standalone publication and would make this already lengthy manuscript unwieldy.

It is unclear what you mean by point #3. Our model cannot be “fit to time series of biomass and catch”. We can assess whether the time series of biomass and catch matches our model output over the same time period, which is what we have done with primary production in Fig 10 (and with timeseries of other taxa in new Figs. 11-12), but we cannot actually fit the model to data, in the same way a statistical model is fit to data.

Additionally, running the old version of the model forward in time would be unlikely able to capture the MHW conditions (which is outside the scope of what we are doing in this manuscript), since such dramatic events occurred (the occurrence of a novel species, for example). That doesn’t mean the earlier model wasn’t useful for the time period in which it was parameterized. The MHW caused such dramatic changes to the ecosystem that we felt it was necessary to rebuild the model, as is presented in this manuscript.

3. A major focus of suggested analyses using this model will be on Chinook salmon. As such, it would be helpful to show some validation to determine how well salmon dynamics are represented in the model. At a minimum, highlighting the Chinook part of the network (e.g., Figures 5 and 6a) could help qualitatively/visually validate and interpret the model.

Thank you for these suggestions. While we agree it would be valuable to have a time series validation of the model for juvenile salmon, we do not currently have a survey with repeat sampling and high temporal resolution to validate the model against. We have now indicated salmon separately within Figs. 5-7 and added a new Fig 13 to make these groups stand out more to the reader, per your suggestions. We agree that these changes have improved the manuscript.

Availability
1. The main model file, code, and a large portion of the model development files are in a public Zenodo repository.

2. The model is more available than many research models that support ecosystem analyses published in peer-review journals. However, I do not believe it meets the criteria of ‘Available’ if it is being published independently as a tool to be used by other researchers and managers. The supporting examples below are the same as listed under Untility_1c:
a. The README.txt file states how there is no workflow to follow from start to finish, some data are not provided due to data ownership criteria, and some files may be missing.
b. The main model file (NCC2_08032022.csv) is available and could be run but if other users wanted to modify the model for different applications, it would be challenging to work through the underlying files and code.
c. The calculations span more than one programming software
d. The “FlowChart.pdf” on Zenodo describes the workflow at a high level but not enough to easily recreate/modify the model.

Thank you for stating that the model is more available than many research models supporting ecosystem analysis – this was certainly our goal. Indeed, it is a difficult process involving 1500 files in our repository, so it makes sense that so many researchers have omitted important details.

While we believe that this manuscript is not just a new tool (see responses above), we disagree with your assessment of whether or not the model is Available. Firstly, there is nothing in the definition of ‘Availability’ from the webpage that isn’t met by our submission:

*If the manuscript’s primary purpose is the description of new software or a new software package, this software must be open source, deposited in an appropriate archive, and conform to the Open Source Definition. If the manuscript mainly describes a database, this database must be open-access and hosted somewhere publicly accessible, and any software used to generate a database should also be open source. If relevant, databases should be open for appropriate deposition of additional data. Dependency on commercial software such as Mathematica and MATLAB does not preclude a paper from consideration, although complete open source solutions are preferred. In these cases, authors should provide a direct link to the deposited software or the database hosting site from within the paper. If the primary focus of a manuscript is the presentation of a new tool, such as a newly developed or modified questionnaire or scale, it should be openly available under a license no more restrictive than CC BY.*

Indeed all of the data and code files to use the model are available, by direct link (within the manuscript) to a long-term repository with a CC BY license. There appears to be some confusion about the distinction between the data/code used to reproduce the work vs the data/code used to actually use the tool – only the latter of which needs to theoretically meet these criteria (although, again, this submission is not only a new tool). We have now clarified the distinction between the reproducibility and model code parts of the data and code submission within our repository. All of the reproducibility data and code we’ve provided, at the same link, is above and beyond what is necessary to make the tool available. We have provided this because we feel strongly about open science and wish to be as transparent as possible, but this is not outlined as a necessity for publication. See individual responses in text above (under Untility_1c) for duplicated points a-d.

Minor Comments
1. Trophic network figures 5&6 are difficult to interpret. A suggestion is to highlight (labels, colored lines, reduced diagrams) some key trophic pathways such as Chinook salmon, killer whales, or other species of commercial/ecological interest.

Thank you for this point. We have now labelled salmon groups distinctly, per your suggestion, for all 3 original network diagrams (Figs 5-7). Additionally, we have added a new Figure 13 that highlights Chinook salmon relationships in a simplified food web diagram. We have made all figure code publicly available such that readers can modify the plots further or if they wish to only see connections to and from other functional groups. We will note that there is something to be gained and something lost in every different view of a network diagram. Reducing the diagram to connections to and from Chinook will highlight more Chinook relationships (Figure 13), but will lose the overall connectivity of the entire network that is more representative of the ecosystem model (Figs 5-7).

2. DietDataSources.csv on Zenodo – many cells reference the “XXXX diet database”; suggest replacing “XXXX” with the name of the database

Thank you for catching this. We have fixed this per your suggestion.

3. Figure 8: Suggest removing the gray grid lines and rotate the axis labels on the x-axis

Thank you for this suggestion. We attempted to make the suggestions that you have proposed, however, we decided to keep the figure as is for a couple of reasons. While removing gray grid lines might look more aesthetically-pleasing, we believe the values of the bars (on both axes) will be much more difficult to decipher without the grid lines to aid the eye (we’re choosing function over aesthetics here). Additionally, rotating the x-axis labels will make most columns twice as wide (1 character width to 2), meaning that the font will have to be decreased to fit the figure onto one page, making the labels illegible.

4. Figure 2 (Cross Shelf Physical Model) is extremely similar to Figure 2 published in Ruzinka et al., 2016. Check for copyright rules and appropriate acknowledgment.

Thank you for this comment. You are right that the figures are similar, but our Figure 2 here was created from scratch in an illustration software. We have now added “adapted from Ruzicka et al 2016” to the figure caption to provide acknowledgement.
