# Supplementary material for: An updated end-to-end ecosystem model of the Northern California Current reflecting ecosystem changes due to recent marine heatwaves
Source: PLoS One. 2024 Jan 19;19(1):e0280366. doi: 10.1371/journal.pone.0280366 (PMC10798527; doi:10.1371/journal.pone.0280366)
Supplement: Attachment — Submitted filename: ResponseToReviewers2.docx [file pone.0280366.s011.docx]

When submitting your revision, we need you to address these additional requirements.

1. Please ensure that your manuscript meets PLOS ONE's style requirements, including those for file naming. The PLOS ONE style templates can be found at 
<https://journals.plos.org/plosone/s/file?id=wjVg/PLOSOne_formatting_sample_main_body.pdf> and 
<https://journals.plos.org/plosone/s/file?id=ba62/PLOSOne_formatting_sample_title_authors_affiliations.pdf>

We confirm that we have conformed to PLOS ONE’s style requirements and uploaded figures according to the PLOS ONE figure naming convention.

2. We note that Figures 1 and 3 in your submission contain map images which may be copyrighted. All PLOS content is published under the Creative Commons Attribution License (CC BY 4.0), which means that the manuscript, images, and Supporting Information files will be freely available online, and any third party is permitted to access, download, copy, distribute, and use these materials in any way, even commercially, with proper attribution. For these reasons, we cannot publish previously copyrighted maps or satellite images created using proprietary data, such as Google software (Google Maps, Street View, and Earth). For more information, see our copyright guidelines: <http://journals.plos.org/plosone/s/licenses-and-copyright>.
We require you to either (1) present written permission from the copyright holder to publish these figures specifically under the CC BY 4.0 license, or (2) remove the figures from your submission:

Figures 1 and 3 were custom made in R with the ggplot2 package. This package accesses boundaries in the public domain from the US Department of Commerce, Census Bureau, Cartographic Boundary Files (https://www.census.gov/programs-surveys/geography/technical-documentation/naming-convention/cartographic-boundary-file.html). Thus, no content within Figures 1 and 3 are copyrighted and the Creative Commons Attribution License (CC BY 4.0) is appropriate for this material. We have now added statements to Figures 1 and 3 noting where the state boundaries came from.

Reviewer #3: This manuscript presents what is essentially an updated version of an existing end-to-end model using the EcoTran platform by Ruzicka et al. (2012) and Ruzicka et al. (2016) in the Northern California Current ecosystem, with several new datasets during and after recent marine heatwaves (2014-2021). The present result showed that the most recent data update is the need to take into account the anomalous oceanographic conditions that occurred during the last decade to facilitate a better understanding of how these conditions can affect the ecosystem, including some vulnerable/threatened species such as the Pacific Salmon in this research. Moreover, with sufficient data, this proves that this model is capable of being used in other places.
So far, the manuscript has been successful, and I have not found any significant flaws or methods. In addition, the documentation of methods and input data is apparent, and all are easily accessible, according to the references in the text. The research result is significant, especially for scientists and managers interested in the NCC ecosystem. However, the authors must carefully examine some minor issues before this manuscript is accepted for publication. Some details in the manuscripts need to recheck to make a better understanding for the reader. Besides, using consistent terms in the publication is essential so the reader is not confused.
Further, I suggest the authors discuss in the discussion part if this model can be applied in the other's location.

Thank you for your positive feedback and for your thoughtful review.

Major issue
None

Minor issue
Line 59: is there any different between marine food web and marine food-web? If there is no different, please remove one of them. NCC change to full name: Northern California Current.

Thank you for this comment. Indeed there is a difference between marine food web and marine food-web. If you search each in Google Scholar, different references are returned. There appears to be some differences in the way that search engines use the additional hyphen. To make our manuscript accessible to either searches, we would prefer to keep both keyword phrases. Keywords are designed to complement the title and abstract – that is, all three are indexed in many search engines. So only words that do not appear in the title or abstract should be added to keywords (keywords are often mis-used in this way). For this reason, “Northern California Current” would not be a useful keyword, since it already appears in the abstract. However, if individuals search “NCC” as a shorthand for “Northern California Current”, they will not find our article without including “NCC” as a keyword (as “NCC” is not included in the abstract).

Line 133: marine heatwaves (MHW)

Thank you for this comment. The acronym “MHW” has been added in this location.

Line 133-134: change to "…..increased magnitude and frequency of marine heatwaves (MHW) (i.e., the 2014-2016 and 2019-2020 MHW) [8–10],"

Thank you for this comment. We have made these changes, per your suggestion.

Line 159: Ruzicka et al. (2012, 2016) [32,33] change to Ruzicka et al. [32,33].. please check again in the whole manuscript for the same format problem (example Line 219, 229 and many more).

Thank you for this comment. We have made these changes in the seven locations we found, per your suggestion.

Line 164: marine heatwaves (MHW) change to MHW

Thank you for this comment. We have made these changes, per your suggestion.

Line 178: Remove Northern California Current and keep the short form NCC. The authors already mentioned the short form before. Also, check the whole manuscript for the same problem (for example, Line 329, 484, etc.).

Thank you for this comment. Because readers do not always read an article sequentially from start to finish, we would prefer keeping the full length of the phrase at the beginning of each section so readers do not have to search for acronym definitions. For example, on line 176 the Methods start, and line 178 is the first time we mention, within that section, the Northern California Current. We believe that defining the acronym within each large section improves the readability of the manuscript. With this said, we replaced Northern California Current with NCC at lines 329, 484, and 865, per your suggestion.

Line 260-262: Can the authors include the P/Q = Production efficiency and AE = Assimilation Efficiency value in Table 1?

Thank you for this comment. The Production efficiency (P/Q) is simply the weight-specific production rate (P/B) divided by the weight-specific consumption rate (C/B), both of which are reported in Table 1. Having an additional column that is easily calculated from the two columns already in the Table, makes it unnecessarily large and cumbersome for the reader and the publication process. We have included this calculation in the Table caption as well as a reference to the csv version of Table 1, where readers can find this information already calculated. Assimilation Efficiency is 0.8 for all consumer groups. Instead of adding another column for this, which again is cumbersome, we have now included it in the Table caption. Readers can find both P/Q and 1 – AE (unassimilated) in the csv version of Table 1 in the supplementary data folder at https://doi.org/10.5281/zenodo.7079777.

Line 286: The Ecopath ecotrophic efficiency (EE) change to The Ecopath EE

Thank you for this comment. We have made these changes, per your suggestion.

Line 292-296: "…… underestimated since many animals can avoid sampling gear"..The term many animals here, including birds or non-aquatic animals? If only aquatic animals, please add "aquatic animals" for better understanding.

Thank you for this comment. Yes this statement includes any animals, including seabirds, which can avoid survey vessels.

Line 309: "wasn't" changed to "was not"

Thank you for this comment. We have made these changes, per your suggestion.

Line 372: the unit for biomass density is not mt/km3, as mentioned in Line 342. Please check the whole manuscript for the unit.

Thank you for this comment. We’ve corrected this to say “areal biomass density”, which is the final unit of biomass density that we use in the model. All volumetric biomass densities mt/km3 (a necessary step for some surveys that provide volume sampled) are converted to areal biomass densities mt/km2 in the model (which some surveys provide directly, see Appendix), so each unit is appropriate. We’ve clarified this throughout the manuscript.

Line 375: Which Appendix do the authors mean? Do you refer to Appendix: Newport Hydrographic Line? I suggest the authors mention more details (such as "see Appendix: Newport Hydrographic Line") because this manuscript has a lot of documents and information. Sometimes make, the reader misunderstands.

Thank you for this comment. We have made these changes throughout the manuscript, per your suggestion.

Line 401: metric tons / km3 change to mt/km3

Thank you for this comment. We have made these changes, per your suggestion.

Line 402: Does the "supplemental code" refer to the file in Ruzicka et al., 2016, or the present study? I can't find the supplemental code at the <https://doi.org/10.5281/zenodo.7079777>. Please check again.

Thank you for this comment. We have now clarified the DOI and directory in the manuscript.

Line 417: Please check the unit = mt km-2

Thank you for this comment. We’ve corrected the formatting to be consistent with other units in the manuscript. The unit itself is correct.

Line 419-420: the full scientific name for E. pacifica and T. spinifera due to first-time mention

Thank you for this comment. We have made these changes, per your suggestion.

Line 422: Change to E. pacifica and T. spinifera

Thank you for this comment. We have made these changes, per your suggestion.

Line 427: Do you mean "Zooplankton, jellies, and pyrosomes" subheading?

Thank you for this question. Yes, we did mean this subheading, and have clarified in the latest version of the manuscript.

Line 432: Again. Please check the subheading name

Thank you for this comment. We have clarified in the latest version of the manuscript.

Line 439 and 457: change to mt/km3

Thank you for this comment. For line 439, mt/km2 is actually the correct unit here. We have now clarified that these biomass densities are by area (areal). We have corrected line 457 to read mt/km3.

Line 460 and 469: the unit for areal biomass densities is not mt/km2? check Line 342

Thank you for this comment. We’ve corrected this throughout the manuscript to say “areal biomass density”, which is the final unit of biomass density that we use in the model (mt/km2). All volumetric biomass densities mt/km3 (a necessary step for some surveys that provide volume sampled) are converted to areal biomass densities mt/km2 in the model (which some surveys provide directly, see Appendix), so each unit is appropriate. We’ve clarified this throughout the manuscript.

Line 484: Northern California Current change to NCC

Thank you for this comment. We have made these changes, per your suggestion.

Line 492 and 494: Ecotrophic Efficiency change to EE.

Thank you for this comment. We have made these changes, per your suggestion.

Line 568 and 577: marine heatwaves change to MHW

Thank you for this comment. We have made these changes, per your suggestion.

Line 628: vertically generalized production model (VGPM) change to VGPM

Thank you for this comment. We have made these changes, per your suggestion.

Line 656: Link, 2010 [39,80]) change to Link [39]. And reference 80 is not published by Link. Please check again the reference.

Thank you for this comment. We have now added “Heymans et al.” to this line to reflect reference 80 (now 81) more accurately.

Line 658: Similar to Line 656. Check the format for reference.

Thank you for this comment. We have made these changes, per your suggestion.

Line 660: biomass-specific production values (P/B) change to P/B

Thank you for this comment. Where we are defining the PREBAL criteria, we would prefer to spell out these names as not everyone is intimately familiar with the acronyms (but keeping the acronyms in parentheses links these terms to the tables and equations in the text).

Line 661: biomass-specific consumption (C/B) change to C/B

Thank you for this comment. Where we are defining the PREBAL criteria, we would prefer to spell out these names as not everyone is intimately familiar with the acronyms (but keeping the acronyms in parentheses links these terms to the tables and equations in the text).

Line 662: ecotrophic efficiency (EE) change to EE

Thank you for this comment. Where we are defining the PREBAL criteria, we would prefer to spell out these names as not everyone is intimately familiar with the acronyms (but keeping the acronyms in parentheses links these terms to the tables and equations in the text).

Line 657-662: I suggest a change to "Food web evaluation criteria guidance from Link [39] states that i; biomass density values of all functional groups should span 5 – 7 orders of magnitude, ii; there should be a 5 – 10% decrease in biomass density (on the log scale) for every unit increase in trophic level, iii; P/B should never exceed C/B values, and iv; EE for each group should be below 1 [39].

Thank you for this comment. It appears that you are suggesting replacing i), ii), iii), and iv) with i;, ii;, iii;, and iv;. We believe using the semicolon in this way is less common and more confusing than the parenthetical as the semicolon often separates clauses in a sentence or objects in a list, whereas the i, ii, iii, and iv here correspond to (and should not be separated from) the following text. For this reason, we are opting to keep the formatting as is.

Line 687: vertically generalized production model (VGPM) change to VGPM

Thank you for this comment. We have made these changes, per your suggestion.

Line 700-702: "Biomass-specific production values (P/B) never exceed biomass-specific consumption (C/B) values, and ecotrophic efficiency (EE) values are all below 1 (Table 1)" change to "P/B never exceed C/B values, and EE values are all below 1 (Table 1)"

Thank you for this comment. Because readers do not always read an article sequentially from start to finish, we would prefer keeping the full length of the phrase at the beginning of each section so readers do not have to search for acronym definitions when starting at any particular section (Results, in this case).

Line 730: "from a vertically generalized production model (VGPM; 2002 - 2021; Fig 10) [49,50]. "change to "from a VGPM 2002 – 2021 (Fig 10) [49,50]."

Thank you for this comment. We have made these changes, per your suggestion.

Line 784: Do the authors mean "see calculated EE in Table 1"

Thank you for this comment. We have made these changes, per your suggestion.

Line 785: euphausiids mean by krills or E. pacifica and T. spinifera or better mentions by the function group in Table 1.

Thank you for this comment. We have now clarified this in the manuscript.

Line 856: marine heatwaves change to MHW

Thank you for this comment. We have made these changes, per your suggestion.

Line 864: Northern California Current change to NCC

Thank you for this comment. We have made these changes, per your suggestion.

Line 881: The data availability statement should mention "supplement or supplement code" because authors used many "see supplement" or "see supplement code" in the text.
Suggestion:
Ecosystem model files, scripts and supplement code, including the balanced and unbalanced diet matrices, biomass estimates, various cleaning scripts, readme files, and all files mentioned in the text can be found at <https://doi.org/10.5281/zenodo.7079777>.

Thank you for this comment. We have made these changes, per your suggestion.

Table and Figure
Check the unit for Figure 4a and 4b.

Thank you for this comment. We can confirm that the unit is correct in Figures 4a and 4b.

Reviewer #4: The present manuscript describes the EcoTran (extension of ECOPATH) ecosystem model parameterized for the Northern Californian Current system. It is an enhanced version of the existing application with the enhancement achieved through defining more functional groups, considering more detailed spatial structure of the model domain, introducing more fisheries, and adding more recent diet data to capture changes in the marine ecosystem with the recent MHW period. The objective of the study is to propose an efficient tool for ecosystem-based fisheries management. For 90 functional groups/species defined in this application, the authors collected and cleaned data on species diet, landings, biomass densities, and fine-tuned specific input parameters to satisfy the mass-balancing equation. Overall, the manuscript addresses complex and timely question, and the extensive effort on data digging, the model parametrization and validation undoubtedly merits being published. However, the outcomes of the study as they are presented now are weak, and the message that the model can serve as a tool suitable for ecosystem analyses and fisheries management is unconvincing. I believe that the current manuscript requires major revision before it could be considered suitable for publication. Several major problems related to the model construction, the outputs and their validation are listed below.

Thank you for your positive feedback, your constructive criticisms, and for your thoughtful review.

The model
No immigration and emigration are considered in the model. It is not explicitly stated, nor discussed in the present manuscript, but according to Ruzicka et al (2012), the modelling “assumed a steady-state system with no biomass accumulation and no migration in or out of the system during the model period”. In Table S3 all emigration parameters are set to 0 and advection accounts only for cross-shelf movements (upwelling and downwelling). The horizontal advection seems to exist only to model nutrient flux. How such assumption can be justified for those highly migratory predators, which migrate seasonally to the NCC domain (e.g., albacore and juvenile bluefin tunas and gray whales), or for smaller pelagic species performing offshore-nearshore movements (jack mackerel), or for the species known to undertake the latitudinal migrations with inter-annual variability (Pacific sardine)?

Thank you for this thoughtful comment. You are correct that immigration and emigration are not included in the model. The original (and continuing) impetus for the NCC model was to study juvenile salmon ecology of threatened and endangered populations of Columbia River fish, and the model domain was thus restricted to the northern section of the California Current. Model development also took advantage of surveys that focused on the Northern California Current. We did not consider latitudinal migration across domain boundaries for two reasons. First, we are unable to directly model the processes that affect migrator groups when they are outside of the model domain during winter months. Inclusion of migration dynamics would require rates and timing of migration to be imposed upon model simulations and assumptions to be made about interannual variability in those processes. We made the simplifying assumption that the trophic processes affecting the migrator groups during the winter season in the south would resemble what those processes would be in the north. This would lead to an overestimate of the impact of migrator groups upon the lower trophic food web during winter months. However, the carry-over bias of over-exploitation of lower-trophic groups by migrators during winter months into the summer is minimized by the high intrinsic productivity and the rapid response of the major lower-trophic groups (i.e., copepods and euphausiids) to the seasonal onset of upwelling in the spring. Second, algorithms to simulate latitudinal migration and intra-domain movement processes are a major effort in and of themselves. They are currently being developed as part of the longer-term improvement of the EcoTran platform, but they are beyond the scope of the present manuscript.

The MHW period is presented as the impetus for updating the model, but it remains unclear whether any notable changes could be captured by the updated model, especially since it has not been compared to its previous version. With respect to the MHW impact, is it at all possible to trace its impact and if yes how, if the key variable, i.e., water temperature, is not accounted for in the model?

Thank you for this thoughtful comment. Water temperature is not necessary to compare ecosystem models between two different states. Models are often compared across years or locations, and a comparison across pre-MHW and post-MHW states would be similar. A subset of us have a paper in review doing exactly this with this model (see: <https://www.biorxiv.org/content/10.1101/2023.08.11.553012v1.full.pdf>, which has now been highlighted in the present manuscript). Additionally, processes are being included into EcoTran through step-by-step improvement – inclusion of water temperature, and how this affects the physiology of various functional groups, is in active development, but beyond the scope of this work.

The model outputs under historical time series of CUTI seem to be highly correlated, just with expected delays between trophic levels. For example, the same pattern is seen in the time series of Market squid (Fig. 11), sardine and anchovy (Fig. 12), while the stock assessments (driven by CPUE and length or age frequency) show very different dynamics. Interestingly, well-documented (see e.g., MacCall et al., 2016) collapse of anchovy is shown by the stock assessment on Fig. 12, but not captured by EcoTran. I’m not an Ecopath expert and familiar with the modeling approach only through the literature, so I’m not sure if this is the problem of the flawed model assumptions, or a particularity of the modeling approach or the ecosystem driven by seasonal upwelling, but the modelled temporal dynamics seems to be simply driven by the dynamics of nitrate and ammonium at the base of the food web. Thanks to the data availability, I could reproduce these plots and trace the pattern down to the base of the food web. Thus, ammonium and shrimp biomass are linearly correlated with Pearson r=0.91 with 2-monthly lag, then shrimp biomass is correlated with anchovy with monthly lag (r=0.88), sardine biomass is correlated with Chinook yearlings with one-year lag (r=0.95), which is correlated with Chinook group with monthly lag (r=0.99), Chinook group is correlated with tunas with 9-monthly lag (r=0.98) etc. Besides, for some species of similar trophic level, the time series dynamics are nearly identical, e.g., for herring, anchovy, mesopelagic fish aggregate, sardine, and squid. Also, surprising correlations exist between higher trophic level species with very different life history traits, e.g., between gray whales and skates&rays group (r=0.9), tuna and seabirds (0.91), or tunas and hake (r=0.94). Such perfect alignment between temporal dynamics of functional groups seems highly unrealistic, indicating an oversimplification, e.g., considering NCC as a closed ecosystem (see my comment above).

“…the modelled temporal dynamics seems to be simply driven by the dynamics of nitrate and ammonium at the base of the food web” – as you say, yes, this is true, but this is what we would expect because it is a bottom-up (upwelling) driven ecosystem. However, the change of local upwelling characteristics (upwelling strength, seasonal timing, upwelling/downwelling event duration) drives local interannual variability, and the effects of these local environmental dynamics are what we are trying to capture in this model. The diets, biomasses, and physiology parameters scale the magnitude and timing (partially dictate the lags), while the upwelling (or other) physical timeseries is what dictates the seasonal patterns and variation to the simulation runs. There are many ecosystem and behavioral complexities that we have certainly not captured. However, we’d argue that our model is still useful as it allows us to understand food web dynamics (e.g., how nutrients/energy stimulated by upwelling propagates up the food web under different conditions).

The fact that complex anchovy dynamics, including widespread population collapse, are not captured by an ecosystem model like this one is not surprising, as predicting such stochastic events are notoriously difficult to capture, especially if we do not fully understand the mechanisms behind such a collapse.

It is not entirely true that the NCC model is a closed system. We are accounting for the daily export losses of phytoplankton and zooplankton production from the NCC domain, and these losses do have a substantial effect on higher trophic levels as has been noted by others, e.g., Botsford et al. 2003 (Fish. Oceanogr. 12:245-259), Rupp et al. 2012 (Fish. Oceanogr. 21:1-19), and Garcia-Reyes et al. 2014 (Prog. Oceanogr. 120:177-188).

Validation
Regarding the phytoplankton validation, the similar periodicity between model predicted phytoplankton biomass density and the VGPM primary production is regarded as the model skill (lines 752-754). However, in many coastal systems, chl-a blooms are driven by upwelling. So, it is very likely that the “remarkably close cyclical resemblance” is primarily the effect of model forcing, i.e., CUTI variability.

Yes, ultimately phytoplankton is driven by the variability in CUTI in the model – as are, ultimately, all functional groups (see detailed response above). However, the remarkably close cyclical resemblance means that we have captured that linkage (CUTI – primary productivity) well enough to reproduce independently observed dynamics. Otherwise, modelled phytoplankton could still be “forced” by CUTI, or any other timeseries, but not have the same timing (e.g., lags, slower or faster periodicity) as the independently observed VGPM timeseries. This alignment is not trivial.

Since all validation plots are shown on standardized y-axis, it is unclear how close the absolute biomass densities are to independent estimates.

Thank you for this point. Yes, we are not intending to be able to predict absolute biomass densities due to the many missing complexities of the ecosystem (see above). There are many hundreds of parameters in this ecosystem model, and we do not expect that they are all perfectly accurate such as to be able to match absolute biomass over time. We believe this to be an unrealistic goal at this moment in the development of ecosystem models. Instead, the goal here is to be able to understand how the food web (or individual functional groups) are affected in a relative way by perturbations to the system (e.g., via CUTI, individual functional group changes, etc.). We do not wish to show absolute biomass densities as we do not think the model should be used in this way; demonstrating such visualizations will lead the reader to believe that it can (or should) be used in this way. Instead, these validation plots are intended to visualize how well the model does (or does not) match temporal trends in surveyed biomass [i.e., how well (or not) does upwelling and our food web parameterization capture real trends].

Finally, if describing the adequate temporal dynamics of intermediate and high trophic level species, is beyond the model capacity, I encourage authors to demonstrate those model skills, for which it can be a helpful and reliable tool for fisheries management. The validation can be done, for example, by running retrospective analyses, results of which can be verified with independent data.

Thank you for this comment. If we understand it correctly, market squid, sardine, anchovy, Pacific mackerel, and jack mackerel are visualized in Figures 11 and 12 as the reviewer describes, and are themselves intermediate trophic level species (Trophic levels = 3.63, 3.11, 3.19, 3.49 and 3.64, respectively). However, as the reviewer points out we do not have any high trophic level species represented in these plots. We agree that this is beyond the model capacity and appreciate the point that it is worth displaying these model skills (or lack thereof). We have now included four more species of relatively high trophic levels (common murres TL = 4.37, sooty shearwaters TL = 4.31, baleen whales TL = 3.69, and southern resident killer whales TL = 5.13) as Figure 13 and we have added language about these plots within the methods and discussion.

Minor comments:
Figures 5-7 are still difficult to read and interpret. Even though a color coding is added to distinguish functional groups, reading the connections between species, and interpreting the diet interactions is impossible. So, either these figures should deliver some qualitative information like footprint and reach in Ruzicka et al. (2012, Figure 6), or it would be better to make group aggregations to make the graph with less vertices, or even provide the diet matrix as in Ruzicka et al. (2012, Table A.3).

Thank you for this comment. We disagree that these figures are impossible to interpret as is. The arrows fade away when interactions are less important and become broader and darker as the interactions are more important. Taking Figure 7 as an example, it is clear that more energy is flowing to functional group 43 than functional group 91. There is something to be gained and something lost in every different view of a network diagram, and we do not intend to be able to capture every angle of it. We believe that reducing the diagram to connections to and from more aggregated groups will lose the overall connectivity of the entire network that we are trying to demonstrate here. We believe this more holistic visualization is more representative of the ecosystem model (Figs 5-7) that we’ve built. We have made all figure code publicly available such that readers can modify the plots further if they wish to only see connections to and from particular functional groups. Changing the line thickness to represent footprint and reach (as in Ruzicka et al. 2012 Fig 6) instead of energy flux won’t actually change the complexity of these figures, it will only change which continuous metric is visualized.

Additionally, as you’ve suggested, we have provided the full diet matrix in the supplementary data (https://doi.org/10.5281/zenodo.7079777) in both the file “NCC2_09032022.csv” (and .xlsx, ‘Diets’ tab) in the root directory and in “Final_DietMatrix_Balanced_Unbalanced_Difference.csv” (and .xlsx) in directory: Reproducibility\DietWork\.

Fig 10. Is there any reason to include all 15 regions into the “time series” of observed phytoplankton, which results in vertical lines every monthly date? Comparing the biomass densities averaged over the model domain seems to be more appropriate. Note also, two months of data are missing in the VGPM PP data file.

Thank you for catching the 2 months of missing data in the VGPM data file. The missing data (August and Sept. 2020) was during active model development and we assume that one iteration of downloading this data included empty values for those months. We have revisited the VGPM data and this has now been fixed in the latest version of the data and code repository. Also, thank you for catching the fact that all 15 regions were plotted unnecessarily. We have now replaced Figure 10 with a similar one with all 15 regions averaged, per your suggestion (which now includes the missing months of data). We agree that this has improved the readability of the figure.

Reviewer #5: a. The model is a useful tool and made available to other researchers to use, with potential to improve ecosystem-based management practices. The extensive data sources and compilation improves the existing model and improves utility for potential users. The authors have addressed previous reviewer comments. I suggest this paper is published and only have minor comments.

Thank you for your positive feedback and for your thoughtful review.

b. Line 234: The latitude of the Newport Hydrographic Line is farther south than 46.7*N. Is this an incorrect latitude, or are the authors referencing a different transect?

Thank you for catching this. This latitude is one of the subregional boundaries and was accidentally copied into the incorrect place. This has now been fixed in the most recent version of the manuscript.

c. Line 772 – 778: In the jelly/gelatinous zooplankton paragraph, the authors could elaborate on the ecosystem and management implications of the rise or persistence of gelatinous organism, as discussed in discussed in Brodeur et al., 2019, not just sampling bias issues.

Thank you for this suggestion. We have now expanded on this discussion, per your suggestion, and we agree that this improves the quality of the manuscript.

d. Line 792-794: Could the authors be more specific in how the six functional groups might improve management, e.g. various stocks versus seasonality?

Thank you for this suggestion. We have now expanded on this discussion, per your suggestion, and we agree that this improves the quality of the manuscript.

e. General comment: In the introduction, the authors note recent changes in the NCC, such as marine heatwaves, ocean acidification, etc. Can you speak in the discussion on if, or how, this model might reflect a regime shift in the ecosystem? How different were the results of this model iteration to previous model iterations (e.g., Ruzicka et al., 2016) that did not include 2015-2019 data?

Thank you for this thoughtful question. We have added language about this to the 6^th^ and to the last paragraph of the discussion. A subset of us have a paper in review that further explores this topic in much more detail (<https://www.biorxiv.org/content/10.1101/2023.08.11.553012v1.full.pdf>). The present manuscript is already quite long and cumbersome as is, but we hope that the sentences that we’ve added here set up the next manuscript in a satisfying way for the reader.
